# Enriched environment improves sevoflurane-induced cognitive impairment during late-pregnancy via hippocampal histone acetylation

**DOI:** 10.1590/1414-431X20209861

**Published:** 2020-08-17

**Authors:** Zhiqiang Yu, Jinxin Wang, Peijun Zhang, Jianbo Wang, Jian Cui, Haiyun Wang

**Affiliations:** 1 Department of Anesthesiology, Tianjin Central Hospital of Gynecology and Obstetrics, Tianjin Key Laboratory of Human Development and Reproductive Regulation, Tianjin, China; 2 Department of Anesthesiology, The Third Central Clinical College of Tianjin Medical University, Tianjin Third Central Hospital, Nankai University Affinity the Third Central Hospital, Tianjin Institute of Hepatobiliary Disease, Tianjin Key Laboratory of Extracorporeal Life Support for Critical Diseases, Tianjin, China

**Keywords:** Cognition, Enriched environment, Histone acetylation, Late-pregnancy, Offspring rat, Sevoflurane

## Abstract

Fetal exposure to sevoflurane induces long-term cognitive impairment. Histone acetylation regulates the transcription of genes involved in memory formation. We investigated whether sevoflurane exposure during late-pregnancy induces neurocognitive impairment in offspring, and if this is related to histone acetylation dysfunction. We determined whether the effects could be reversed by an enriched environment (EE). Pregnant rats were exposed to 2.5% sevoflurane or control for 1, 3, or 6 h on gestational day 18 (G18). Sevoflurane reduced brain-derived neurotrophic factor (BDNF), acetyl histone H3 (Ac-H3), and Ac-H4 levels and increased histone deacetylases-2 (HDAC2) and HDAC3 levels in the hippocampus of the offspring on postnatal day 1 (P1) and P35. Long-term potentiation was inhibited, and spatial learning and memory were impaired in the 6-h sevoflurane group at P35. EE alleviated sevoflurane-induced cognitive dysfunction and increased hippocampal BDNF, Ac-H3, and Ac-H4. Exposure to 2.5% sevoflurane for 3 h during late-pregnancy decreased hippocampal BDNF, Ac-H3, and Ac-H4 in the offspring but had no effect on cognitive function. However, when the exposure time was 6 h, impaired spatial learning and memory were linked to reduced BDNF, Ac-H3, and Ac-H4, which could be reversed by EE.

## Introduction

Fetal brain development is sensitive to drugs and the *in-utero* environment during pregnancy. Emergency non-obstetric or fetal surgeries may occur during gestation; however, the effects of general anesthetics on the neurodevelopment and cognition of the offspring is not well understood. The United States Food and Drug Administration (FDA) issued a warning against repeated or prolonged (>3 h) exposure to certain general anesthetic agents during pregnancy (1), but clinical evidence is limited, and the reasons for this warning were not elaborated on in detail.

Sevoflurane is commonly used in obstetric anesthesia. Animal studies have shown that exposure of pregnant rats to sevoflurane may induce long-term memory impairment in the offspring. The effects of sevoflurane on the neurodevelopment and cognition of the offspring depends on the exposure concentration, time, and frequency. Most studies have focused on middle pregnancy (2,3), while only a few studies have focused on late pregnancy (4,5). The brain and central nervous system are vulnerable to anesthetic agents in late pregnancy, when synapse formation, dendritic arborization, and cortical lamination occur (6). However, the effects on the offspring when mothers are exposed to a single clinically relevant concentration of sevoflurane during late pregnancy remain unclear.

Histone acetylation, a common form of epigenetic modification, is critical for the regulation of learning and memory, which is regulated by histone acetyltransferases (HATs) and histone deacetylases (HDACs) (7). There is evidence that dysregulation of Ac-H3 and Ac-H4 in the hippocampus contributes to reduced memory function by inhibiting critical learning and memory genes (8). Epigenetic mechanisms are gradually being recognized as regulators of gene transcription in long-term memory processes that are associated with the level of HDACs (9). Knockdown of HDAC2 and HDAC3 in the mouse hippocampus has been shown to increase mRNA expression of brain-derived neurotrophic factor (BDNF) and improve memory formation (10). General anesthetics cause epigenetic modifications, such as induced histone-3 hypoacetylation in rats (P7), which results in downregulation of BDNF transcription and the induction of long-term impairments in neuronal development (11).

An enriched environment (EE) is an effective and simple method to improve cognitive impairment. Moderate daily exercise induces histone hyperacetylation in the rat brain and swimming ameliorates isoflurane-induced memory impairment in neonatal mice by enhancing hippocampal histone acetylation (12), an effect similar to that of HDAC inhibitors.

In this study, we investigated the relationship between dysregulation of histone acetylation and memory deficits in the offspring of rats exposed to sevoflurane during late-pregnancy, and if EE would ameliorate cognitive impairment by regulating histone acetylation.

## Material and Methods

### Animals and anesthesia

**Figure 1:**
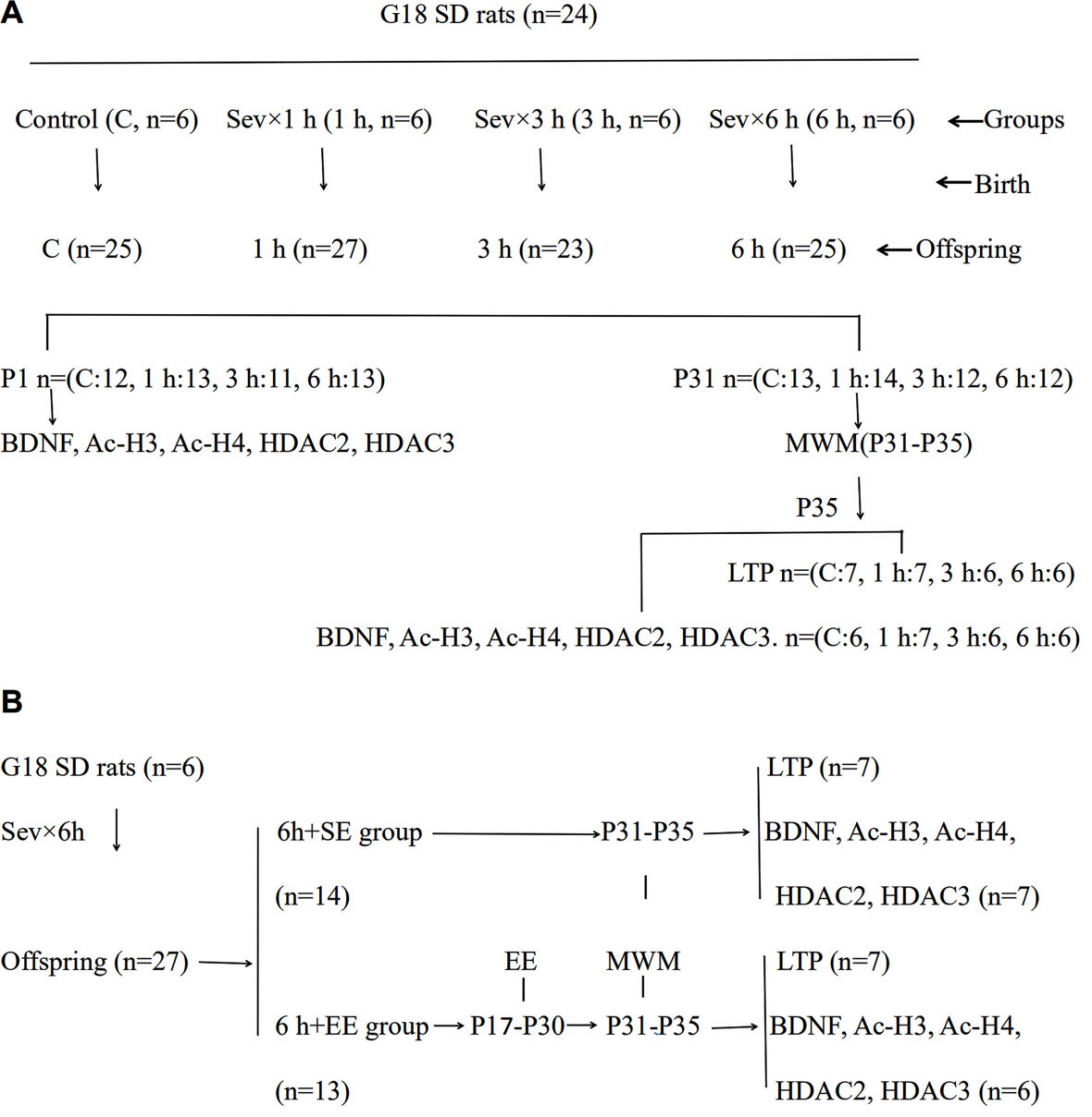
Flowchart of the (**A**) first and (**B**) second experimental protocols. G18: gestational day 18; P1-P35: postnatal days 1 to 35; MWM: Morris water maze; Sev: 2.5% sevoflurane; EE: enriched environment; SE: standard environment.

The study protocol adhered to the Regulations for Research by the Tianjin Medical University Committee, and was approved by the Tianjin Medical University Animal Care Committee on the Use of Animals in Research and Teaching. Twenty-four pregnant rats with gestational age of 18 days (G18) were randomly assigned to the control group or anesthesia group (Figure 1A). One minimum alveolar concentration (MAC) of sevoflurane is usually used in human obstetric anesthesia. In humans, one MAC is equivalent to approximately 1.76%, and in rats one MAC is approximately 2.5%. Therefore, we decided that 2.5% sevoflurane is a clinically relevant concentration. Rats received 2.5% sevoflurane in 50% oxygen for 1, 3, or 6 h (Sev×1, Sev×3, and Sev×6 groups, respectively) in an anesthetizing chamber (Datex-Ohmeda Inc., USA). In the control group, rats were exposed to 50% oxygen. The concentrations of oxygen, sevoflurane, and arterial blood gas were evaluated continuously. Following termination of anesthesia, the rats recovered in 50% oxygen. All pregnant rats were housed until delivery. Neonatal rats were randomly assigned to one of two arms in every group; hippocampal tissue of neonatal rats was harvested for biochemistry studies in one arm on postnatal day 1 (P1), while other neonates were raised to 31 days in the other arm. The Morris water maze (MWM) was used for behavioral studies for 5 days from P31. Hippocampal tissue was then harvested on P35 for biochemistry and patch studies. All rats were euthanized under pentobarbital anesthesia.

### Western blot analysis

Hippocampal tissue from P1 and P35 offspring were prepared for determination of BDNF, Ac-H3, Ac-H4, HDAC2, and HDAC3 levels. Glyceraldehyde-3-phosphate dehydrogenase (GAPDH) expression was used for the normalization of western blots. Protein levels were measured by incubation with antibodies against BDNF (1:1000; ab203573; Abcam, UK), anti-acetylhistone H3 (1:1,000; ab61231; Abcam), anti-acetylhistone H4 (1:1000; ab114146, Abcam), anti-HDAC2 (1:1000; ab75602; Abcam), anti-HDAC3 (1:1000; ab209840; Abcam), and GAPDH (1:1000; 6004-1-1g; Abcam). Protein bands were visualized by enhanced chemiluminescence and quantified with ImageJ software (NIH, USA).

### Morris water maze

In this study, the MWM was used to evaluate spatial learning and memory in offspring. A round pool (diameter, 160 cm; height, 60 cm) was filled with 20°C water to a height of 1.5 cm below the top of a 12-cm diameter removable cylindrical platform. The pool was covered with a curtain and was placed in a separate room with visual cues on every wall of the pool. The offspring rats were trained in the MWM four times per day from P26 to P30 and were tested four times per day from P31 to P35. Each of the offspring rats was put randomly into the water, facing the wall of the pool, to search for the platform, and the starting locations differed for each rat. When a rat found the platform within 90 s, it was allowed to stay on the platform for 20 s, and the period taken to find the platform was defined as the escape latency. If a rat did not find the platform within 90 s, it was gently guided to the platform and allowed to rest for 20 s. In this case, the escape latency was recorded as 90 s. At the end of the reference training (P35), the platform was removed, and the rat was released in the opposite quadrant where they were allowed to swim for 90 s; the frequencies at which the rat crossed the platform area was recorded as the number of platform crossings. A video tracking system (Shanghai Mobile Datum Ltd., China) recorded the swimming speed, escape latency, platform crossing times, and the time spent in each quadrant(s) of offspring. The data were analyzed using motion-detection software for the MWM (Institute of Materia Medica, Chinese Academy of Medical Sciences & Peking Union Medical College, China). After every trial, the rat was placed in a cage warmed with a heat lamp until dry before being returned to its cage.

### Extracellular field potential recordings

Hippocampal slices from tissue harvested on P35 were prepared for electrophysiological studies. Field evoked postsynaptic potentials (fEPSPs) were recorded in the cornu ammonis 1 (CA1) region. Brain slices were placed in a recording chamber and perfused (1.5-2.0 mL/min) with artificial cerebrospinal fluid (ACSF), which was bubbled with 95%O_2_/5%CO_2_. ACSF-filled theta-glass stimulating pipettes and recording pipettes were put in Schaffer collaterals and the CA1 region accordingly. The distance between the stimulating and recording electrodes was 400-500 μm. fEPSPs were elicited once every 30 s and recorded at 10 kHz (Molecular Devices, USA). Input/output curves were created, and high frequency stimulation (HFS, 100 Hz) was adjusted to evoke 50-60% of the maximum fEPSP. To induce long-term potentiation (LTP), HFS was applied. The fEPSP slope was recorded for 20 min before and 50-60 min after LTP induction. Data were analyzed with Clampfit 10.2 software (Molecular Devices).

### Exposure to enriched environment

Six rats (G18) were exposed to 2.5% sevoflurane for 6 h. To determine whether exposure to an EE would ameliorate sevoflurane’s neurotoxic effects, offspring from another Sev×6 group were exposed to either a standard environment (SE) or an EE. MWM, greater dendritic spine densities, and LTP were observed in the Sev×6+EE and Sev×6+SE groups (Figure 1B).

Offspring were exposed to EE daily for two weeks from P17 to P30 for 6 h (between 10 am to 4 pm). Thirteen rats were put in a large cage (720×550×300 cm) containing differently shaped objects: 2 shelters, 3 wheels, 2 ladders, 1 plastic tunnel, 1 trapeze, 3 balls, and 5 toys in number varying from 1 to 5. The location of objects and toys were changed every two days. Food and water were available *ad libitum*. EE cages provided enough scope for motor, visual, somatosensory, and cognitive stimulation. The SE was the same cage without the equipment.

### Statistical analysis

Data are reported as means±SD. All analyses were performed with SPSS 19.0 software (IBM, USA). The differences among experimental groups regarding BDNF, Ac-H3, Ac-H4, HDAC2, HDAC3, swimming speed, platform crossing time, escape latency, and the time spent in each quadrant(s) were analyzed using two-way analysis of variance (ANOVA) followed by Tukey’s test. A P value <0.05 was considered statistically significant.

## Results

### Maternal exposure to sevoflurane induced changes in BDNF, Ac-H3, Ac-H4, HDAC2, and HDAC3 in the hippocampus of offspring

**Figure 2:**
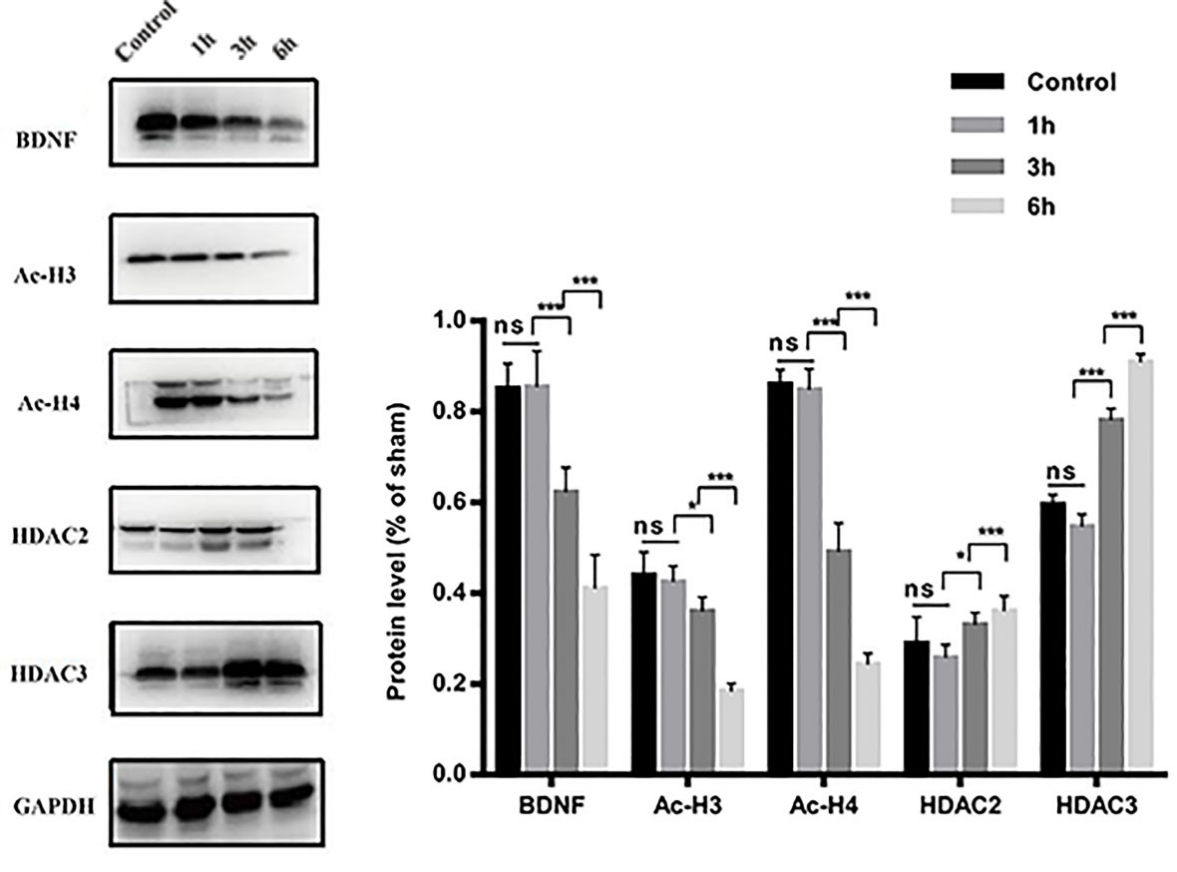
Effects of 2.5% sevoflurane exposure during late pregnancy on postnatal day 1 offspring rats. Exposure to 2.5% sevoflurane for 3 and 6 hours in late-pregnancy rats decreased Ac-H3, Ac-H4, BDNF, and increased HDAC2 and HDAC3 in the hippocampus. Compared with the 3-h group, the levels of Ac-H3, Ac-H4, and BDNF were reduced, and the levels of HDAC2 and HDAC3 were increased in the 6-h group. Data are reported as means±SD. *P<0.05, ***P<0.001 (ANOVA followed by Tukey’s test). ns: not significant.

**Figure 3:**
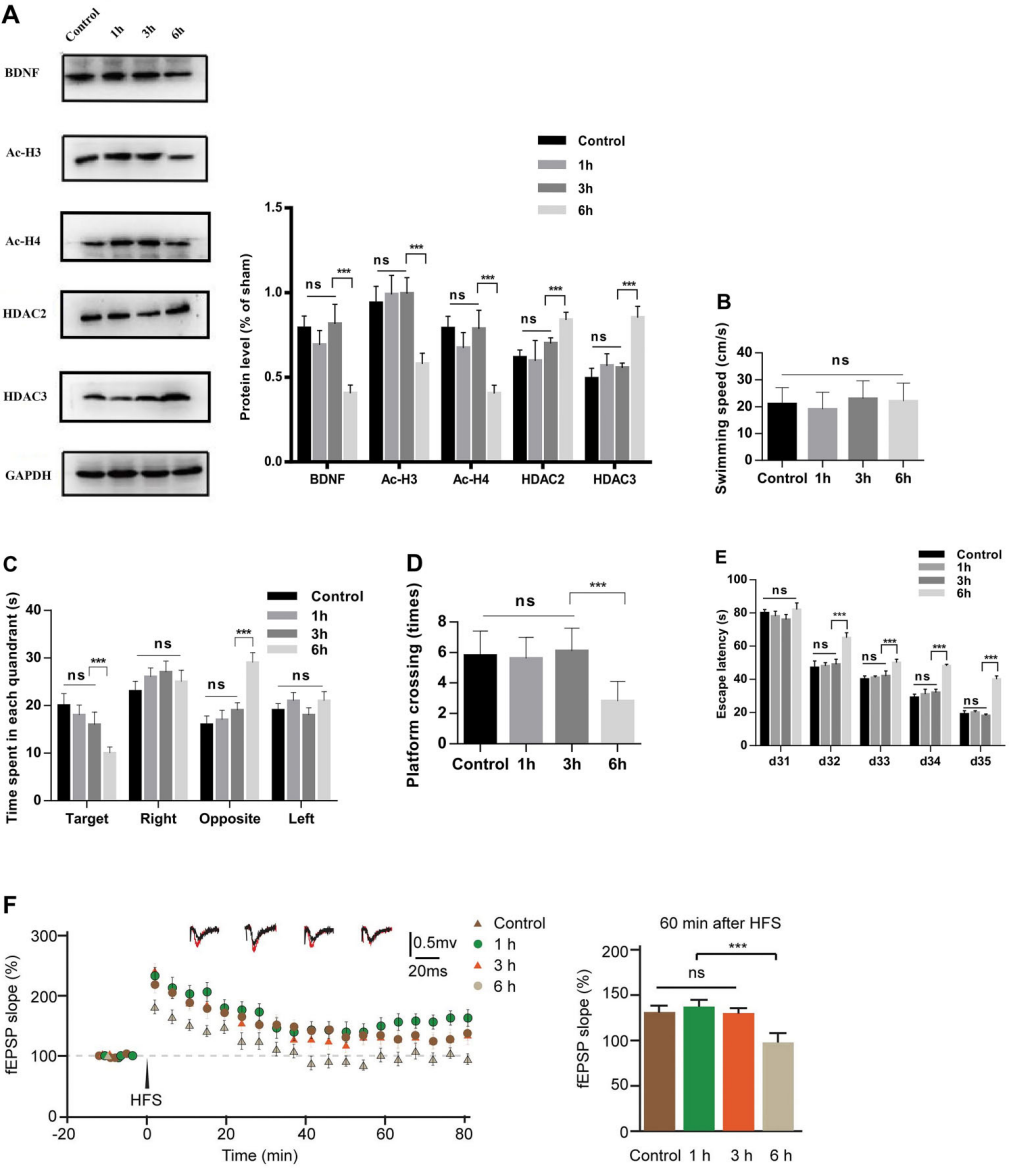
Effects of 2.5% sevoflurane exposure during late pregnancy on postnatal day 35 offspring rats. **A**, Exposure to 2.5% sevoflurane in late-pregnancy rats decreased BDNF, Ac-H3, and Ac-H4 and increased HDAC2 and HDAC3 in the hippocampus of offspring in the 6-h group. **B**, There were no significant differences in swimming speed. **C**, In the 6-h group, the time spent in the target quadrant was decreased and the time in the opposite quadrant was increased. **D**, Platform crossing times were decreased and **E**, escape latency was extended. **F**, Long-term potentiation measured in field evoked postsynaptic potentials (fEPSP) was inhibited after high frequency stimulation (HFS). Data are reported as means±SD. ***P<0.001 (ANOVA followed by Tukey’s test). ns: not significant, d: day: EE: enriched environment; SE: standard environment.

On P1, maternal exposure to sevoflurane decreased the levels of Ac-H3 (F=62.99, P<0.001), Ac-H4 (F=248.1, P<0.001), and BDNF (F=99.19, P<0.001) and increased the levels of HDAC2 (F=9.576, P=0.0009) and HDAC3 (F=260.4, P<0.001) in the hippocampus of the Sev×6 group compared with the other three groups. Sev×3 reduced Ac-H3 (F=7.424, P=0.0106), Ac-H4 (F=149.9, P<0.001), and BDNF (F=32.12, P<0.001) and increased HDAC2 (F=4.228, P=0.0467) and HDAC3 (F=124.8, P<0.001) compared with Sev×1 and control group. There was no significant difference between the control group and the Sev×1 group (Figure 2; P>0.05). On P35, Ac-H3 (F=27.24, P<0.001), Ac-H4 (F=28.10, P<0.001), and BDNF (F=46.80, P<0.001) levels were reduced and HDAC2 (F=13.68, <0.001) and HDAC3 (F=87.32, P<0.001) were increased in the hippocampus in the Sev×6 group, although there was no significant difference between the control, Sev×1, and Sev×3 groups (Figure 3A; P>0.05).

### Maternal exposure to sevoflurane induced impairment in spatial learning and memory of offspring

The MWM was used to assess cognition in the offspring from P31 to P35. There was no significant difference in swimming speed (Figure 3B; P>0.05) or the time spent in the right and left quadrants (P>0.05). The time spent in the target quadrant was significantly decreased (F=17.82, P<0.001) and the time spent in the opposite quadrant was significantly increased (F=37.67, P<0.001; Figure 3C). Platform crossing times were significantly decreased (F=38.8, P<0.001; Figure 3D), and the escape latency was significantly increased (F_32_=47.53, F_33_=22.87, F_34_=52.89, F_35_=155.7; P<0.001; Figure 3E) in the Sev×6 group compared with the other groups. There were no significant differences in escape latency, platform crossing times, or the time spent in the target and opposite quadrant among the control, Sev×1, and Sev×3 groups (P>0.05).

### Maternal exposure to sevoflurane reduced LTP in the hippocampal CA1 region of offspring

The magnitude of LTP induced by HFS was decreased in the Sev×6 group compared to that in the other three groups (F=13.64, P<0.001; Figure 3F). No difference was observed in the magnitude of LTP (the mean fEPSP slope during the last 20 min of recording) induced by HFS in the control, Sev×1, and Sev×3 groups (P>0.05).

### Sevoflurane exposure-induced impairment in offspring brain was ameliorated by an enriched environment

**Figure 4:**
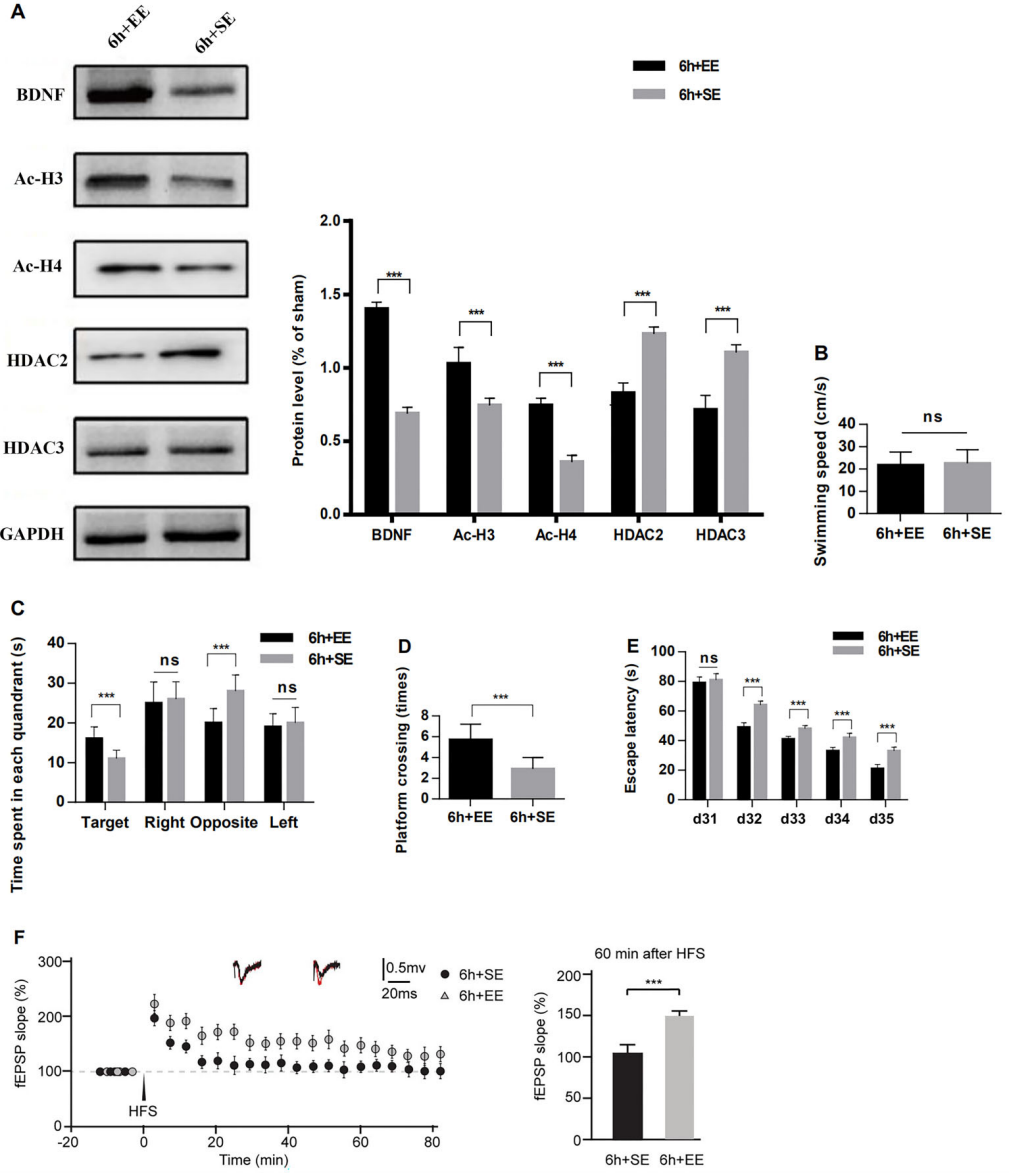
Enriched environment (EE) ameliorated impairment in long-term potentiation (LTP), spatial learning, and memory of postnatal-day-35 offspring rats induced by sevoflurane exposure compared to standard environment (SE). **A**, Compared to the 6-h group, EE increased BDNF, Ac-H3, and Ac-H4 and reduced HDAC2 and HDAC3. **B**, There was no significant difference in swimming speed. **C**, The time spent in the target quadrant was increased and the time in opposite quadrant was decreased. **D**, Platform crossing times were increased and **E**, escape latency was decreased. **F**, Long-term potentiation measured in field evoked postsynaptic potentials (fEPSP) was enhanced in the 6-h+EE group. ***P<0.001 (t-test). ns: not significant.

Compared to the Sev×6+SE group, EE increased BDNF (F=749.4, P<0.001), Ac-H3 (F=46.65, P=0.001), and Ac-H4 (F=256.9, P<0.001) and reduced HDAC2 (F=98.26, P=0.0002) and HDAC3 (F=161.5, P<0.001) expression in the hippocampus on P35 (Figure 4A). There was no significant difference in swimming speed between groups (P>0.05; Figure 4B). The time spent in the target quadrant was increased and the time spent in the opposite quadrant was decreased (Figure 4C), platform crossing times were significantly increased (F=147.2, P<0.001; Figure 4D), and the escape latency (P<0.001) was significantly reduced in the Sev×6+EE group compared to the Sev×6+SE group (Figure 4E). The magnitude of LTP induced by HFS was increased in the Sev×6+EE group compared to that in the Sev×6+SE group (F=33.95, P<0.001; Figure 4F). There were no differences in these results between male and female offspring (P>0.05).

## Discussion

The present study demonstrated that a 6-h exposure to 2.5% sevoflurane in late pregnancy led to impairment in spatial learning and memory in offspring, which was associated with reduced Ac-H3, Ac-H4, and BDNF, and increased HDAC2 and HDAC3 expression in the hippocampus. We found that EE exposure improved cognitive impairment in the offspring, and increased histone acetylation and BDNF expression in the hippocampus.

Abnormal histone acetylation is a key factor in the pathogenesis of cognitive impairment. Sevoflurane-exposed neonatal rats have been shown to have reduced levels of Ac-H3 and Ac-H4 and increased levels of HDAC3 and HDAC8 at P33 and P50, and these abnormalities are normalized by HDAC inhibitors (13). HDACs negatively regulate long-term memory processes (14) by increasing the expression of certain HDACs, which impairs spatial, contextual, and object recognition memories mediated by the hippocampus. BDNF is one of the most well-studied neurotrophins and exerts neurotrophic and neuroprotective functions in brain development. Decreased Ac-H3 and Ac-H4 in the hippocampus has been shown to reduce BDNF expression, impair key BDNF signaling pathways, and induce cognitive function deficits in rats (15). This study demonstrated that exposure of rats to 2.5% sevoflurane for 3 or 6 h during late pregnancy led to a reduction of Ac-H3, Ac-H4, and BDNF, and increased hippocampal levels of HDAC2 and HDAC3 at P1. The hippocampus is an important brain region for learning and memory; thus, abnormal expression of histone acetylation and BDNF in the neonate may induce long-term cognitive deficits.

Histone acetylation is widely implicated in synaptic plasticity and memory formation in the CA1 area. Ac-H3 and Ac-H4 levels are commonly used to assess histone acetylation homeostasis in the central nervous system. Histone acetylation activates transcription, which can be reversed by HDACs. HDAC2 negatively regulates structural and functional synaptic plasticity as well as memory formation in the hippocampus (16). BDNF increases the number of inhibitory and excitatory synapses, promotes synapse formation, participates in LTP, and is related to learning and memory (17). LTP is the neuronal basis of learning and memory (18), and the MWM is a classical and reliable method to evaluate spatial learning and memory in animal models (19–21). In the present study, we found that when rats were exposed to 2.5% sevoflurane for 3 h during late pregnancy, no differences in the expression of Ac-H3, Ac-H4, HDAC2, HDAC3, or BDNF were observed in their offspring at P35. These results differed from those observed in offspring at P1, and there were no differences in LTP or the MWM test at P35 in the offspring of sevoflurane-exposed rats compared to the control group. However, when the exposure time was up to 6 h, Ac-H3, Ac-H4, and BDNF were decreased, while HDAC2 and HDAC3 were increased, offspring learning and memory function was reduced at P35, and LTP was inhibited. The neurotoxic effects of sevoflurane on the fetus depend on exposure concentration, duration, and gestational age. Therefore, we propose that the exposure of rats to sevoflurane for 3 h during late pregnancy caused dysfunction of histone acetylation in the P1 offspring, but the effect was short-term, and these adverse changes were reversible and would recover after development. Moreover, these changes would not impair cognitive function in juvenile rats. In addition, long-term damage to the cognitive function of the offspring may be linked to dysfunction of histone acetylation and abnormal BDNF levels only with prolonged sevoflurane exposure.

There are many highly reproducible rodent findings regarding exposure of the developing brain to general anesthetics; however, compared to humans, rodent brain development is substantially shorter, and the complexity of neuronal networks is obviously different. The relationship between neuron development and the length of general anesthesia exposure is very complex and extrapolating what sevoflurane exposure in rats means to humans cannot be done by simple mathematical modeling. Therefore, we cannot rely exclusively on rodent data if we are to make inroads into understanding the potential relevance of animal data to humans (22).

EE plays an important role in the improvement of post-stroke cognitive impairment, improves MWM performance, and increases hippocampal LTP; it has been suggested that the mechanism underlying these effects may involve histone acetylation (23). EE has been shown to restore the inhibition of LTP induced by MK-801 in the hippocampal-medial prefrontal pathway in adult rats (24). Moreover, accumulating evidence indicates that EE increases the expression of neurotransmitter receptors and neurotrophic factors, promotes dendritic branching, and enhances learning and memory processes (25,26); however, the exact mechanisms by which EE reduces the neurotoxicity of sevoflurane remain to be determined, and the developmental outcomes of fetal exposure to sevoflurane may be influenced by postnatal environmental factors.

BDNF is a trophic factor associated with cognitive improvement and a previous study suggested that prolonged exercise increases the expression of β-hydroxybutyrate, induces the activity of the BDNF gene promoter, and decreases the binding of HDAC2 and HDAC3 to the BDNF promoter in the mouse hippocampus, causing an increase in BDNF release (27). In this study, we found the expression of Ac-H3, Ac-H4, and BDNF were increased, while levels of HDAC2 and HDAC3 were decreased in the hippocampal CA1 region, and the impairment of learning and memory ability in juvenile rats was alleviated by EE. Therefore, we speculate that EE improved the impairment in learning and memory induced by sevoflurane by enhancing histone acetylation and BDNF protein expression in the hippocampus.

There are several limitations to this study. First, we only observed the effects of EE in the Sev×6 group and did not investigate its effects in either control or unanesthetized offspring groups. Second, we carried out behavioral tests on male and female offspring; however, a previous study has indicated sex differences in the vulnerability to anesthetics (28). Third, histone acetylation is regulated by HATs and HDACs, but we did not assess HATs and histone acetylases. Further, while HDACs have numerous subtypes, we only examined Ac-H3, Ac-H4, HDAC2, and HDAC3. Fourth, we did not determine whether sevoflurane decreased histone acetylation directly or indirectly by inhibiting HDACs. Finally, rodent and human brain development differs; thus, further study is required to confirm the relationship between histone acetylation and cognition in humans.

The present study demonstrated a mechanism by which exposure to a clinically relevant concentration of sevoflurane for 3 h in late pregnancy rats caused histone acetylation and a reduction in BDNF in the neonatal hippocampus, and the abnormality recovered with normal development. However, a 6-h exposure led to impairment in spatial learning and memory with a reduction in histone acetylation and BDNF in juvenile rats. EE improved the sevoflurane-induced cognitive impairments by enhancing hippocampal histone acetylation and BDNF expression.
